# Glasgow coma scale-pupils at 24-hours as a reliable marker for extremely poor prognosis in traumatic brain injury – A prospective cohort

**DOI:** 10.1016/j.clinsp.2026.101047

**Published:** 2026-07-23

**Authors:** Pedro Fortes Osório Bustamante, Roberta Muriel Longo Roepke, Filipe Matheus Cadamuro, Francisco Falleiros de Mello, Bárbara Vieira Carneiro, Brasil Chian Ping Jeng, Wellingson Silva Paiva, Sérgio Henrique Bastos Damous, Edivaldo Massazo Utiyama, Estevão Bassi

**Affiliations:** a Department of Surgery, Division of General Surgery and Trauma, Hospital das Clinicas HCMFMUSP, Faculdade de Medicina, Universidade de Sao Paulo, Sao Paulo, SP, Brazil; b D'Or Institute for Research and Education (IDOR), Rio de Janeiro, RJ, Brazil; c Intensive Care Unit, A.C. Camargo Cancer Center, São Paulo, SP, Brazil; d Intensive Care Unit, Hospital Israelita Albert Einstein, São Paulo, SP, Brazil; e Department of Neurosurgery, Hospital das Clinicas, Faculdade de Medicina, Universidade de Sao Paulo, Sao Paulo, SP, Brazil; f Intensive Care Unit, Hospital Alemão Oswaldo Cruz, São Paulo, SP, Brazil

**Keywords:** Traumatic brain injury, Glasgow coma scale pupils score, Neuroprognostication, Brain Death, Intensive Care

## Abstract

**Objective:**

Early prognosis determination after traumatic brain injury remains challenging, yet reliable early markers remain limited. A Glasgow Coma Scale-Pupil (GCS-P) score of 1, combining a GCS of 3 and bilaterally nonreactive pupils, has been proposed as a marker of catastrophic injury, although some patients may recover. The authors hypothesized that persistence of a GCS-P score of 1 after 24 h could predict poor prognosis.

**Methods:**

Descriptive study in a quaternary ICU in Brazil, including adult patients admitted between 2019 and 2025 with a pre-sedation GCS of 3 and bilaterally nonreactive pupils. Patients with unknown initial neurological status or a Marshall Computed Tomography Classification < 3 were excluded. Clinical characteristics, imaging findings, treatments, and outcomes were prospectively collected. The primary outcome was the Glasgow Outcome Scale-Extended (GOSE) at hospital discharge.

**Results:**

Among 2645 ICU admissions, 868 were due to TBI, and 49 met the inclusion criteria. Patients were predominantly young (median age 38-years), male (79.6%), and had high illness severity (median SAPS3 of 68). Most suffered blunt trauma, and nearly half underwent hemicraniectomy. All patients who persisted with a GCS-P score of 1 after 24-hours died during hospitalization (n = 36, 95% CI 90.3%–100%). Among those who improved their GCS-P score within 24-hours (n = 13), mortality was 54% (95% CI 25.1%–80.8%), and 31% (95% CI 9.1%–61.4%) recovered consciousness (GOSE > 2).

**Conclusion:**

Persistence of a GCS-P score of 1 after 24-hours is a highly reliable marker of poor prognosis in patients with severe TBI. These findings warrant external validation.

## Highlights

• Simple bedside neurologic marker informs early neuroprognostication.
• Persistence of GCS-P = 1 at 24 h predicted 100% in-hospital mortality.
• Early improvement in GCS-P identified patients with potential neurologic recovery.
• Findings support aggressive early care before prognostic reassessment.

## Introduction

Traumatic Brain Injury (TBI) is a major health concern, associated with high mortality and significant long-term morbidity among survivors, especially in low and middle-income countries.^1^, ^2^, ^3^, ^4^ Despite advances in primary prevention strategies, the global incidence of TBI is projected to rise in the coming years. Patients presenting with a Glasgow Coma Scale (GCS) score of 8 or lower are classified as having severe TBI and are at increased risk for prolonged hospital stay and worse prognoses.^5^^,^^6^

Identifying patients who won’t have good neurological outcomes, despite being a high interest subject, is challenging, as many patients improve even years after the injury.^7^, ^8^, ^9^, ^10^ Pupillary reactivity might reflect third cranial nerve paralysis caused by intracranial hypertension, and the presence of bilaterally nonreactive pupils at ICU admission is considered a moderately reliable predictor of poor functional outcome.^5^

The GCS Pupils score (GCS-P) combines the GCS with pupillary reactivity.^11^, ^12^, ^13^ A GCS-P of 1, representing patients with a GCS of 3 and bilaterally nonreactive pupils, has been proposed as a potential marker of catastrophic injury. However, some patients with this score may still experience meaningful neurological recovery.^14^ Possible explanations include peripheral causes of nonreactive pupils or the efficacy of an early aggressive treatment in preventing irreversible damage.

The authors hypothesized that the persistence of a GCS-P score of 1 after 24-hours of initial care might be a definitive marker of poor prognosis. This study aims to assess whether the GCS-P at 24-hours is a reliable marker of unfavorable outcomes in patients with TBI.

## Material and methods

### Study design, setting and ethics

This is a descriptive study in one ICU in a quaternary academic hospital in São Paulo, Brazil, that admits mainly major trauma and neurocritical patients. The authors included data from January 2019 to September 2025. Because of the study design ‒ retrospective analysis of a prospective quality improvement database, the application of informed consent was waived by the institutional review board (CAAE: 61,006,622.8.0000.0068).

### Study population and outcomes

The authors included all adult patients admitted to the ICU with a reported GCS of 3 before the use of sedatives, and bilaterally nonreactive pupils. The authors excluded patients with an unknown GCS or pupillary status at the scene or at admission, and those with a Marshall Computed Tomography Scoring System of 1 or 2, since in these cases, nonreactive pupils are unlikely to be attributable to intracranial hypertension.^15^

The main outcome of interest was the Glasgow Outcome Scale Extended (GOSE), an eight-point scale ranging from 1 (death) to 8 (upper-good recovery),^16^^,^^17^ at hospital discharge. Secondary outcomes included hospital death, ICU and hospital length of stay, and dichotomized GOSE (good outcome: GOSE 3‒8; bad outcome: GOSE 1‒2).

### Data collection and variables

The characteristics of the patients, pupillary response to light at admission and on the next day, injury mechanism, presence of other injuries in addition to TBI, presence of vascular (carotid or vertebral) lesions, use of organic support, surgical procedures, and invasive monitoring of intracranial pressure were collected prospectively. ICU and hospital outcomes, the decision to withhold or withdraw life-sustaining therapies, as well as the time of the decision, were also recorded prospectively.

Pupillary response was assessed with the use of a pen torch or light, without the use of a pupillometer. The Marshall Computed Tomography Scoring System was recorded by an intensivist with experience in trauma care. The Glasgow Outcome Scale at hospital discharge was collected retrospectively using Electronic Health Records (EHR). When the information was missing from the EHR, no imputation was made. All data were collected through a secure web application (RedCap).^18^^,^^19^

### Statistical analysis

According to the descriptive design of the study, no sample calculation and no comparisons were made. Categorical variables are presented according to occurrence and percentages; normally distributed variables are presented as mean and standard deviation. Variables with nonnormal distributions are presented as medians and interquartile intervals.

Descriptive analyses were performed for the overall cohort and stratified according to the GCS-P status after 24-hours, categorized as “GCS-P = 1 at 24h” and “GCS-P > 1 at 24h”. The distribution of the GOSE at hospital discharge was described for each group, and exact 95% Confidence Intervals for each GOSE category were calculated using the Clopper-Pearson method. Secondary outcomes were also reported as proportions with exact 95% Confidence Intervals.

To evaluate the association between extracranial traumatic injuries and neurological outcome, a multivariable ordinal logistic regression model was fitted with GOSE at hospital discharge as the dependent variable. Independent variables included the presence of traumatic injuries involving the face, spine, thorax, abdomen, and extremities. Adjusted odds ratios with 95% Confidence Intervals were calculated. Because of complete separation, pelvic trauma was excluded from the multivariable model.

A post-hoc sensitivity analysis excluding patients with limitations of life-sustaining treatment was performed. The distribution of GOSE at hospital discharge and corresponding confidence intervals was recalculated in this restricted cohort.

Data were processed and analyzed using Python free software, pandas, and stats models libraries.^20^, ^21^, ^22^

## Results

Between January 2019 and September 2025, a total of 2645 patients were admitted to the ICU, of whom 868 were admitted due to TBI. Fifty-two patients had a GCS-P score of 1. Of these, three had a Marshall Computed Tomography Classification of 1 or 2 and were excluded, leaving 49 patients for the final analysis.

### Patients’ characteristics

Table 1 summarizes the characteristics of the 49 included patients. The median age was 38-years (IQR 32–47), and nearly 80% were male (39/49). They presented with high acute illness severity, reflected by a median SAPS3 score of 68 (IQR 62–79), and were previously healthy (median Charlson Comorbidity Index of 0). Most patients suffered blunt trauma; motor-vehicle collisions (34.7%, 17/49) were the most common trauma mechanism, followed by high-energy falls (> 3 m; 22.4%, 11/49).

**Table 1:** General characteristics of patients.

Baseline characteristics	
Age (years)	38 [32 – 47]
Male gender, n (%)	39 (79.6%)
Non-caucasian, n (%)	25 (51%)
Caucasian, n (%)	24 (49%)
SAPS3 at ICU admission	68 [62 – 79]
SOFA on the 1st day of ICU	11 [10 – 13]
Charlson Comorbidity Index	0 [0–0]
**Injury mechanism**	
Penetrating	2 (4.1%)
* Firearm injury*	2 (100%)
Blunt trauma	47 (95.9%)
* Road traffic injuries*	20 (42.5%)
* High-energy fall (> 3m)*	11 (23.4%)
* Low-energy fall (< 3m)*	6 (12.8%)
* Other blunt trauma*	10 (21.3%)
**Marshall CT scoring scale at ICU admission**	
3	4 (8.2%)
4	6 (12.2%)
5	25 (51%)
6	14 (28.6%)
**CT findings at admission**	
Subarachnoid hemorrhage	41 (83.7%)
Diffuse edema	39 (79.6%)
Subdural haematoma	31 (63.3%)
Epidural haematoma	14 (28.6%)
Diffuse axonal injury	7 (14.3%)
Contusion	23 (47%)
**Concomitant injuries on admission**	
Exclusively TBI	15 (28.8%)
Face	20 (40.8%)
Spinal cord	19 (38.8%)
Thoracic	21 (42.9%)
Abdominal	8 (16.3%)
Pelvic	6 (12.2%)
Extremities	18 (36.7%)
Arterial lesion (carotid or vertebral)	5 (13.2%)
Cardiac arrest at presentation	8 (16.3%)
**Surgical procedures**	
Any neurosurgical procedure	39 (79.6%)
* Hemicraniectomy*	24 (49%)
* External Ventricular Drainage*	25 (51%)
* ICP monitorization*	25 (51%)
* Epidural heamatoma treatment*	5 (10.2%)
* Subdural heamatoma treatment*	26 (47%)
* Cerebral contusion treatment*	8 (16.3%)
* Depressed skull fracture treatment*	2 (4.1%)
Non-neurological surgery	13 (26.5%)

CT, Computed Tomography; ICP, Intracranial Pressure; ICU, Intensive Care Unit; TBI, Traumatic Brain Injury.

### Lesions characteristics and surgical procedures

The most frequent cranial Computed Tomography (CT) findings were traumatic subarachnoid hemorrhage in 84% (41/49) and diffuse cerebral edema in 80% (39/49). A Marshall CT score of 5 or 6 was present in 51% and 28.6% of patients (25/49 and 14/49), respectively.

TBI was the only injury in nearly 30% (15/49) of patients. Concomitant thoracic, facial, and spinal/vertebral injuries were present in 43%, 41%, and 39% (21/49, 20/49, and 19/49), respectively. Arterial injuries were identified in 13% (5/49), and 16% (8/49) of the patients experienced cardiac arrest at the trauma scene.

Almost 80% (39/49) of patients underwent neurosurgical interventions, with hemicraniectomy performed in 49% (24/49). Intracranial pressure monitoring was used in 51% (25/49). All neurosurgical interventions were performed as early as possible after evaluation of the neurosurgeon, prior to ICU admission, and before the 24-hour assessment. Furthermore, 26% (13/49) underwent non-neurological surgical procedures.

### Outcomes

All patients who remained with a GCS-P score of 1 24-hours after admission died during hospitalization, most meeting criteria for brain death (Table 2). Four patients in this group were discharged from the ICU, but had prolonged hospital stay and ultimately died in the hospital ward after 48-, 49-, 132-, and 238-days.

**Table 2:** Outcomes at Hospital Discharge and length of stay.

Primary Outcome – GOSE at hospital discharge	GCS-P = 1 at 24h (n = 36)	GCS-P > 1 at 24h (n = 13)
GOSE 1	36 [90.3 – 100%]	7 [25.1 – 80.8%]
GOSE 2	0 [0 – 9.7%]	2 [1.9 – 45.4%]
GOSE 3	0 [0 – 9.7%]	3 [5 – 53.8%]
GOSE 4	0 [0 – 9.7%]	1 [0.2 – 36%]
**Secondary outcomes**		
In-hospital mortality	36 [90.3 – 100%]	7 [25.1 – 80.8%]
Brain death	27 [57.8 – 87.9%]	3 [5 – 53.8%]
GOSE 1‒2	36 [90.3 – 100%]	9 [38.6 – 90.9%]
ICU LOS, days	2.55 [1.65 – 4.15]	22.46 [5.32 – 48.1]
Hospital LOS, days	3 [2 – 5]	44 [6 – 62]

ICU, Intensive Care Unit; GCS-P, Glasgow Coma Scale Pupil Score; GOSE, Glasgow Outcome Scale Extended; LOS, Length of Stay.

Among the 13 patients who showed improvement in GCS-P within the first 24-hours, scores ranged from 2 to 8, with a median of 3. Two patients improved their GCS but remained with bilaterally nonreactive pupils and subsequently died in the hospital. Overall hospital mortality in this group was 54% (7/13). Recovery of consciousness (GOSE > 2) occurred in 31% of these patients (4/13). Individual components and outcomes of the GCS-P at 24-hours after admission are presented in Table 3.

**Table 3:** GCS-P component description of patients with GCS-P > 1 at 24-hours.

Patient	GCS at 24 h^a^	Pupil status at 24 h	GCS-P at 24 h	Neurosurgical intervention within 24 h	GOSE at hospital discharge
1	3	Anisocoric, 1 reactive pupil	3	SHT	1
2	4	Bilaterally nonreactive	2	No intervention	1
3	3	Both reactive	3	ICP, EVD	1
4	3	Anisocoric, 1 reactive pupil	2	SHT, EHT, DSFT	1
5	3	Both reactive	3	ICP, EVD, CCT	4
6	3	Anisocoric, 1 reactive pupil	2	HC, ICP, EVD, SHT, CCT	3
7	8	Both reactive	8	HC, ICP, EVD, SHT, CCT	3
8	3	Both reactive	3	HC, ICP, EVD, SHT, CCT	1
9	3	Both reactive	3	HC, ICP, EVD, SHT	3
10	3	Both reactive	3	SHT, EHT, ICP, EVD	2
11	3	Anisocoric, 1 reactive pupil	2	ICP, EVD	2
12	3	Anisocoric, 1 reactive pupil	2	HC, ICP, EVD, SHT	1
13	5	Bilaterally nonreactive	3	HC, SHT	1

a Improvement in the Glasgow Coma Scale score was entirely driven by changes in the motor component. CCT, Cerebral Contusion Treatment; DSFT, Depressed Skull Fracture Treatment; EHT, Epidural Heamatoma Treatment; EVD, External Ventricular Drainage; GCS, Glasgow Coma Scale; GCS-P, Glasgow Coma Scale Pupil Score; GOSE, Glasgow Outcome Scale Extended; HC, Hemicraniectomy; ICP, Intracranial Pressure Monitorization; SHT, Subdural Heamatoma Treatment.

In the multivariable analysis (Table S1), none of the evaluated extracranial injuries were independently associated with GOSE at hospital discharge. Although facial and spinal cord injuries showed a trend toward lower GOSE categories, and thoracic and extremity injuries showed a trend toward higher GOSE categories, these associations did not reach statistical significance.

All patients in both groups required invasive mechanical ventilation and vasopressor support, as expected given the severity of their neurological injury. Limitation of life-sustaining therapies was instituted in 11% (4/36) of patients with a GCS-P score of 1 at 24-hours, at 5-, 41-, 114-, and 180-days after admission, and in 15% (2/13) of those with a GCS-P score > 1 at 24-hours, at 4- and 7-days. Organ support use and limitation of life-sustaining therapies are detailed in Table 4. A post-hoc sensitivity analysis excluding those submitted to the limitation of life-sustaining therapies yielded similar results (Supplement Table S2).

**Table 4:** Organ support use and limitation of life-sustaining therapies.

	GCS-P = 1 at 24h (n = 36)	GCS-P > 1 at 24h (n = 13)
**Organ support use**		
Vasopressor	36 (100%)	13 (100%)
RRT	0	2 (15.4%)
MV	36 (100%)	36 (100%)
Tracheostomy	4 (11.1%)	7 (53.8%)
**Any definition of limiting life-sustaining therapies**	4 (11.1%)	2 (15.4%)
**Time to first decision of limitation after admission, days**	78 [32 – 131]	5 [5 – 6]

GCS-P, Glasgow Coma Scale Pupil Score; MV, Mechanical Ventilation; RRT, Renal Replacement Therapy.

## Discussion

### Statement of main findings

This study describes the outcomes of TBI patients with the most severe early prognostic marker ‒ a GCS-P score of 1 ‒ treated in a quaternary hospital in Brazil. Nearly 87% of these patients died during hospitalization. Furthermore, every patient who remained with a GCS-P score of 1 for at least 24-hours after admission died in the hospital. To our knowledge, this is the first study to identify the persistence of this early marker as a highly reliable indicator of poor prognosis in TBI patients.

### Relationship with the literature

The value of the GCS-P score as a prognostic marker has been evaluated by other authors. Lin et al. analyzed discrimination and calibration of GCS-P in a cohort of 4372 neurocritical patients.^12^ Mahajan et al. evaluated whether GCS-P could outperform GCS in determining hospital mortality and functional outcomes at 6 months in a cohort of 573 TBI patients.^13^ Ambesi et al. analyzed whether GCS-P could be a better predictor of hospital mortality than GCS in a cohort of almost 400 TBI patients.^11^ Unlike these prior assessments of GCS-P, this study evaluates the prognostic significance of the worst stratum of the score, the GCS-P of 1, and its persistence after a defined resuscitation window of 24-hours.

The overall survival rate in the present cohort (12%) is comparable to previous reports involving patients with severe TBI and poor early neurological markers. Tien et al. evaluated 104 patients presenting with a GCS-P score of 1 and found a 100% mortality rate.^23^ Chamoun et al. analyzed 189 patients with an initial GCS of 3 and reported a hospital survival of 51%^24^ ; however, when focusing on those with bilateral fixed and dilated pupils, only 20% survived. Jamous et al. evaluated 21 patients with a GCS-P score of 1 and observed 100% mortality within 30-days.^25^ Tian et al. evaluated a cohort restricted to patients with a GCS-P score of 1 who underwent decompressive craniectomy and reported a 36% survival rate, but this design may have excluded individuals with immediate criteria for brain death.^14^ In the present cohort, all patients admitted to the ICU were included, including those being evaluated for brain death or supported as potential organ donors. Notably, among survivors in Tian's study, half remained unconscious at hospital discharge, and the 6-month survival rate was only 25%. The present contemporary cohort reinforces these findings and, additionally, demonstrates the critical importance of the 24-hour reassessment.

Unlike previous studies primarily focused on determining whether patients with a GCS-P score of 1 at admission have any chance of meaningful recovery and whether aggressive care is justified, the main objective was to evaluate the subgroup of patients who remained with a GCS-P score of 1 after the initial 24-hours of resuscitation and treatment. The high progression to brain death (75%) in this subgroup suggests that persistence of this marker may also serve as an early identifier of potential organ donors, facilitating family discussions about donation.

### Strengths and limitations

This study has several strengths. Data were prospectively collected using a tool specifically designed for this purpose, minimizing information bias and ensuring good-quality data for analysis. Bilateral fixed pupils may result not only from intracranial hypertension but also from peripheral or ocular injuries; therefore, the authors excluded patients with a Marshall Computed Tomography Classification of <3 to reduce heterogeneity and ensure that an absent pupillary response was more likely attributable to an intracranial pathology. Indeed, one of the three excluded patients ultimately had a favorable outcome. Decisions to limit life-sustaining therapies were recorded and relatively infrequent early in the course of critical illness, mitigating the risk of a self-fulfilling prophecy ‒ a major concern in neuroprognostication research.^26^

This study also has limitations. Long-term outcomes were not evaluated, which could underestimate recovery in a small subset of patients. However, since all patients who persisted with a GCS-P score of 1 died in the hospital, this limitation does not affect the primary conclusion. The GOSE score was assigned retrospectively based on electronic medical records, which may introduce errors; to reduce this, the authors reviewed documentation from physicians, nurses, and physical therapists. Given the extremely high mortality rate, the impact of this limitation is likely small. Another limitation is the high frequency of early deaths and brain death diagnoses, which could suggest an initial conservative management; however, nearly 80% of patients underwent neurosurgical procedures, and half underwent hemicraniectomy, supporting the interpretation that early mortality reflected the extreme severity of injury rather than therapeutic nihilism.

Early limitations of life-sustaining therapies could potentially influence the results, as patients with the potential for improvement might not receive full treatment. In the present study, however, despite the high initial severity, only 12% of patients were subject to decisions to limit life-sustaining therapies. In all cases, at least three days of full critical care support were provided, as recommended.^5^ Furthermore, in half of these patients, such decisions were made at least one month after the initial treatment, suggesting that therapeutic nihilism was unlikely to have influenced the present results.

Extracranial injuries were present in nearly 70% of patients; these injuries may contribute to secondary injury and potentially influence outcomes, thereby limiting the interpretation of GCS-P as an isolated prognostic marker; to explore this possibility, a post hoc analysis was performed to address this issue and no statistical significance was found; however, due to limited sample size and the relatively low prevalence of some extracranial injuries, wide confidence intervals and reduced statistical power prevented accurate interpretation of this association.

Finally, this is a single-center study, and the generalizability of these findings to other settings warrants caution.

### Implications for practice and research

**Fig. 1:**
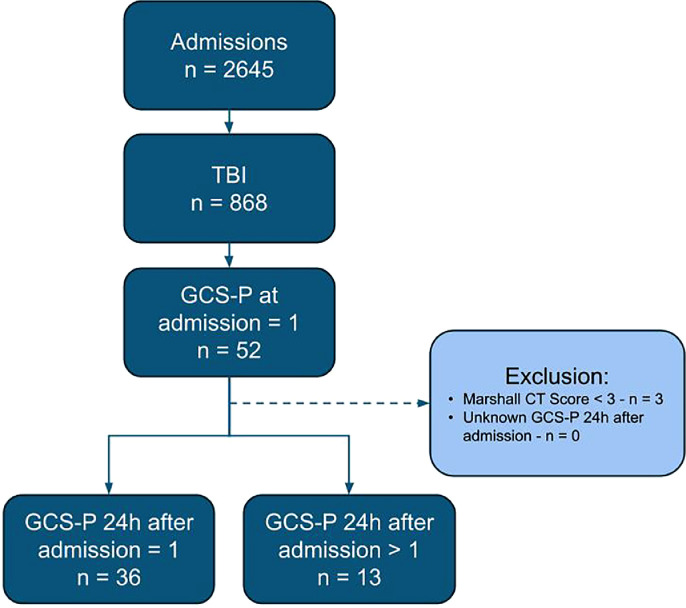
Flow of patients in the study. CT, Computed Tomography; GCS, Glasgow Coma Scale; GCS-P, Glasgow Coma Scale Pupil Score; TBI, Traumatic Brain Injury.

This cohort confirms the extremely high mortality of TBI patients presenting with a GCS-P score of 1 and identifies the persistence of this score after 24-hours as a potential early marker of near-certain mortality. Conversely, the presence of neurologic improvement within the first 24-hours ‒ and the observation of meaningful recovery among some of these patients ‒ underscores that a GCS-P of 1 on admission should not be used in isolation to limit therapy and the importance of aggressive early management when there are no clear signs of brain death or futility (Fig. 1).

After aggressive surgical and clinical treatment, if pupillary reactivity does not return after the first day, clinicians should communicate the profoundly poor prognosis to families and consider shifting goals of care toward comfort-focused management or organ-donor support when appropriate.

These findings should be validated in multicenter cohorts to assess their applicability, exclude center-specific effects on outcomes, and to confirm the generalizability of these findings.

## Conclusions

The present findings indicate that maintaining a GCS-P score of 1 at 24 h is a highly reliable marker of extremely poor prognosis in TBI patients, supporting its use as an early prognostic indicator.

## Details page

The authors meet all the criteria for authorship and confirm that this manuscript has not been published elsewhere and is not under consideration by another journal.

## Ethics

This study was approved with a waiver of informed consent by the institutional review board (CAAE: 61,006,622.8.0000.0068).

## Reporting checklist

This manuscript is fully compliant with STROBE guidance for reporting observational studies. STROBE checklist is included in the submission.

## Declaration of generative AI and AI-assisted technologies in the manuscript preparation process

The primary author declares that ChatGPT (GPT‑4, OpenAI) was used solely for grammatical revision and to provide suggestions for improving the clarity and flow of the text. No part of the content was automatically generated, and the final version of the manuscript is the sole responsibility of the author. The final version of this manuscript was approved by all authors.

## Authors’ contributions

Pedro Fortes Osório Bustamante: Contributed to the conception of the study, was responsible for data collection, drafted the first version of the manuscript, and critically revised and approved the final manuscript.

Roberta Muriel Longo Roepke: Contributed to the conception of the study, designed the data collection tool, participated in data collection, and critically revised and approved the final manuscript.

Filipe Matheus Cadamuro: Contributed to the conception of the study and critically revised and approved the final manuscript.

Francisco Falleiros de Mello: Contributed to data collection and critically revised and approved the final manuscript.

Bárbara Vieira Carneiro: Critically revised and approved the final manuscript.

Chian Ping Jeng: Critically revised and approved the final manuscript.

Wellingson Silva Paiva: Critically revised and approved the final manuscript.

Sérgio Henrique Bastos Damous: Critically revised and approved the final manuscript.

Edivaldo Massazo Utiyama: Critically revised and approved the final manuscript.

Estevão Bassi: Contributed to the conception of the study and critically revised and approved the final manuscript.

## Funding

This research did not receive any specific grant from funding agencies in the public, commercial, or not-for-profit sectors.

## Data availability

The datasets generated and/or analyzed during the current study are available from the corresponding author upon reasonable request.

## Declaration of competing interest

The authors declare no conflicts of interest.

## Appendix Supplementary materials

Image, application 1
