# Supplementary material for: Glasgow coma scale-pupils at 24-hours as a reliable marker for extremely poor prognosis in traumatic brain injury – A prospective cohort
Source: Clinics (Sao Paulo). 2026 Jul 23;81:101047. doi: 10.1016/j.clinsp.2026.101047 (PMC13427555; doi:10.1016/j.clinsp.2026.101047)
Supplement: Supplementary file 1 [file mmc1.docx]

**CLINICS-D-26-00200**

**Supplementary Material**

Supplement to: Glasgow Coma Scale-Pupils at 24 hours as a reliable marker for extremely poor prognosis in traumatic brain injury – a prospective cohort.

**Table S1** Multivariable ordinal logistic regression analysis evaluating the association between extracranial traumatic injuries and GOSE at hospital discharge.

| **Concomitant Injury** | **Adjusted OR^a^** | **95% CI** | **p-value** |
| --- | --- | --- | --- |
| Face | 0.132 | 0.011 – 1;579 | 0.110 |
| Spinal cord | 0.272 | 0.022 – 3.376 | 0.311 |
| Thoracic | 2.690 | 0.229 – 31.607 | 0.431 |
| Abdominal | 0.528 | 0.031 – 9.043 | 0.659 |
| Extremities | 4.316 | 0.409 – 45.580 | 0.224 |

^a^ Odds Ratios represent the odds of being in a higher GOSE category.

CI, Confidence Interval; OR, Odds Ratio.

**Table S2** Sensitivity analysis excluding patients with limitation of life-sustaining therapy.

| **Sensitivity analysis – GOSE at hospital discharge** | **GCS-P=1 at 24h (n=32)** | **GCS-P >1 at 24h (n=11)** |
| --- | --- | --- |
| GOSE 1 | 32 [89.1 – 100%] | 5 [16.7 – 76.6%] |
| GOSE 2 | 0 [0 – 10.0%] | 2 [2.3 – 51.8%] |
| GOSE 3 | 0 [0 – 10.9%] | 3 [6 – 61%] |
| GOSE 4 | 0 [0 – 10.9%] | 1 [0.2 – 41.3%] |

GCS-P, Glasgow Coma Scale Pupil Score; GOSE, Glasgow Outcome Scale Extended.
